# Management of Tricuspid Valve Regurgitation During Surgical Ventricular Restoration for Ischemic Cardiomyopathy

**DOI:** 10.21470/1678-9741-2023-0013

**Published:** 2023-07-18

**Authors:** Amr A. Arafat, Fatimah Alhijab, Monirah A. Albabtain, Juan Alfonso, Abdullah Alshehri, Huda Ismail, Adam I. Adam, Claudio Pragliola

**Affiliations:** 1 Adult Cardiac Surgery Department, Prince Sultan Cardiac Center, Riyadh, Riyadh, Saudi Arabia; 2 Cardiothoracic Surgery Department, Tanta University, Tanta, Egypt; 3 Cardiology Clinical Pharmacy Department, Prince Sultan Cardiac Center, Riyadh, Riyadh, Saudi Arabia; 4 Cardiac Research Department, Prince Sultan Cardiac Center, Riyadh, Riyadh, Saudi Arabia

**Keywords:** Tricuspid Valve, Tricuspid Valve Insufficiency, Patient Readmission, Conservative Treatment

## Abstract

**Introduction:**

We studied the effect of tricuspid valve (TV) surgery combined with surgical ventricular restoration (SVR) on operative outcomes, rehospitalization, recurrent tricuspid regurgitation, and survival of patients with ischemic cardiomyopathy. Additionally, surgery was compared to conservative management in patients with mild or moderate tricuspid regurgitation. To the best of our knowledge, the advantage of combining TV surgery with SVR in patients with ischemic cardiomyopathy had not been investigated before.

**Methods:**

This retrospective cohort study included 137 SVR patients who were recruited from 2009 to 2020. Patients were divided into two groups - those with no concomitant TV surgery (n=74) and those with concomitant TV repair or replacement (n=63).

**Results:**

Extracorporeal membrane oxygenation use was higher in SVR patients without TV surgery (P=0.015). Re-exploration and blood transfusion were significantly higher in those with TV surgery (P=0.048 and P=0.037, respectively). Hospital mortality occurred in eight (10.81%) patients with no TV surgery vs. five (7.94%) in the TV surgery group (P=0.771). Neither rehospitalization (log-rank P=0.749) nor survival (log-rank P=0.515) differed in patients with mild and moderate tricuspid regurgitation in both groups. Freedom from recurrent tricuspid regurgitation was non-significantly higher in mild and moderate tricuspid regurgitation patients with no TV surgery (P=0.059). Conservative management predicted the recurrence of tricuspid regurgitation.

**Conclusion:**

TV surgery concomitant with SVR could reduce the recurrence of tricuspid regurgitation; however, its effect on the clinical outcomes of rehospitalization and survival was not evident. The same effects were observed in patients with mild and moderate tricuspid regurgitation.

## INTRODUCTION

**Table:** 

Abbreviations, Acronyms & Symbols				
BMI	= Body mass index		ICD	= Implantable cardioverter defibrillator
CABG	= Coronary artery bypass grafting		ICU	= Intensive care unit
CCS	= Canadian Cardiovascular Society		LV	= Left ventricular
CI	= Confidence interval		MI	= Myocardial infarction
CPB	= Cardiopulmonary bypass		MR	= Mitral regurgitation
CRRT	= Continuous renal replacement therapy		NYHA	= New York Heart Association
ECMO	= Extracorporeal membrane oxygenation		PASP	= Pulmonary artery systolic pressure
EDD	= End-diastolic diameter		PCI	= Percutaneous coronary intervention
EDV	= End-diastolic volume		PPM	= Permanent pacemaker
EDVi	= End-diastolic volume index		RBC	= Red blood cell
EF	= Ejection fraction		RV	= Right ventricular
ESD	= End-systolic diameter		RWMA	= Regional wall motion abnormality
ESV	= End-systolic volume		SHR	= Sub-distributional hazard ratio
ESVi	= End-systolic volume index		SVR	= Surgical ventricular restoration
EuroSCORE	= European System for Cardiac Operative Risk Evaluation		TR	= Tricuspid regurgitation
HR	= Hazard ratio		TV	= Tricuspid valve
IABP	= Intra-aortic balloon pump			

Surgical ventricular restoration (SVR) is the intended procedure to correct the abnormal geometrical alterations following myocardial ischemia^[1]^. Ischemic cardiomyopathy may result in several changes, including abnormal spherical rather than elliptical shape, increased ventricular size, and reduced ventricular function^[2]^. Restoring the normal anatomical shape, reducing the size of the enlarged ventricle, revascularization, and treatment of valvulopathies would be targeted following ischemia. Some studies proposed the importance of achieving normal left ventricular (LV) volume by SVR rather than focusing on improving ejection fraction (EF) alone by revascularization procedures^[3]^. The importance of SVR procedures in patients with ischemic cardiomyopathy or heart failure has become a great concern, especially in nations where heart transplantation is still limited or less frequently performed^[1,4]^.

SVR was usually performed following anterior myocardial infarction with consequent LV end-systolic volume index > 60 ml/m^2^ ^[5]^. Several techniques have evolved, and further studies are recommended to understand better the effectiveness and long-term outcomes of the different SVR procedures. Prucz et al. conducted a study to compare the effects of combining SVR with coronary artery bypass grafting (CABG) *vs.* CABG alone in patients with ischemic cardiomyopathy and enlarged ventricular size. The study showed less rehospitalization in patients with SVR and CABG and better improvements in New York Heart Association (NYHA) class^[6]^. Another study showed a decrease in mitral regurgitation (MR) grade by combining SVR and CABG^[7]^. Few studies discussed the impact of combining mitral surgery with SVR. Castelvecchio et al. found that early and mid-term outcomes of combining SVR and mitral repair can be predicted according to angina symptoms before surgery^[8]^.

However, combining SVR and tricuspid valve (TV) surgery was not explored earlier. SVR patients are high-risk patients^[9]^, and it is unknown whether adding additional TV intervention with prolonged operative and cardiopulmonary bypass times could lead to improved immediate and long-term outcomes. In this study, we aimed to investigate the effect of combining TV surgery with SVR on operative outcomes and long-term cardiac rehospitalization, recurrent tricuspid regurgitation (TR), and survival of patients with ischemic cardiomyopathy. Additionally, we compared TV surgery *vs.* conservative management in patients with mild or moderate TR.

## METHODS

### Study Design

We conducted a retrospective cohort study including 137 patients who underwent SVR for ischemic cardiomyopathy from November 2009 to October 2020. Patients were divided according to the TV procedure into two groups: SVR without TV surgery (n=74) and SVR with TV repair or replacement (n=63). Approval of the study was obtained from the Research Committee of the Cardiac Center (IRB approval No: R20043). The need for patient consent was waived.

### Study Data and Outcomes

Preoperative data were collected according to European System for Cardiac Operative Risk Evaluation (EuroSCORE) II definitions^[10]^. Concomitant CABG and mitral valve surgery were reported. Study outcomes were hospital complications and long-term freedom from cardiac rehospitalization, recurrent TR, and survival. Surgical techniques used for SVR in our center were reported before by Calafiore et al.^[11]^. TV repair was performed using the DeVega technique (n=9), MC3™ rigid ring (Edwards Lifesciences, Irvine, California, United States of America) (n=1), and SMN50 flexible band (Sovering MiniBand, SMN50, Sorin, Saluggia, Italy) (n=51). For patients who had TV replacement, tissue valves were used (n=2). The patients were followed clinically after discharge for one and six months, then at yearly intervals, and the closing follow-up date was May 2020.

### Echocardiography

All patients had transthoracic echocardiograms before surgery and at discharge. A total of 495 echocardiography examinations were available for all patients during a 10-year follow-up. Changes in EF, pulmonary artery systolic pressure (PASP), and right ventricular (RV) dilatation were reported and compared between groups.

### Statistical Analysis

Analysis was performed using Stata 16.1 (Stata Corp, College Station, Texas, United States of America). Continuous variables were tested for normality and compared with the *t*-test or Mann-Whitney U test. Categorical data were compared with the Chi-squared test or Fisher’s exact test. Data were presented as mean and standard deviation for normally distributed continuous variables or median (25^th^ 75^th^ percentiles) for non-normally distributed continuous variables. Non-continuous data were presented as counts and percentages. A *P*-value < 0.05 was considered statistically significant.

The Kaplan-Meier curve was used for survival distribution. Multivariable Cox regression with backward elimination was used to identify factors affecting survival. The entry *P*-value was 0.1, and the stay *P*-value was 0.05.

Fine and Gray method was used to perform competing risk regression^[12]^. Death was considered a competing risk for recurrent TR and cardiac rehospitalization. Choosing the final model of multivariable competing regression was performed in the same method as Cox regression.

Random effect regression was used to compare the change in EF and PASP over time between both groups. Random effect ordinal logistic regression was used to compare the change in the degree of RV dilatation.

## RESULTS

### Preoperative Data

Patients who had TV surgery were significantly younger (61.77±9.21 *vs.* 57.77±9.90 years; *P*=0.015). Most patients were male (65 [87.84%] *vs.* 49 [77.78%]; *P*=0.168 in patients without and with TV surgery, respectively). Patients with TV surgery had significantly higher EuroSCORE II (*P*=0.012) and higher NYHA class III-IV (*P*=0.003). There were no differences in diabetes mellitus, atrial fibrillation, myocardial infarction, or history of percutaneous coronary interventions between the groups (Table 1).

**Table 1:** Comparison of the preoperative patient characteristics between surgical ventricular restoration patients with or without concomitant tricuspid valve surgery.

	No TV surgery (n=74)	TV surgery (n=63)	*P*-value
Age (years)	61.77±9.21	57.77±9.90	0.015
BMI (Kg/m^2^)	26.98 (24.23-30.09)	26.71 (23.83-30.4)	0.961
Male sex	65 (87.84%)	49 (77.78%)	0.168
EuroSCORE II	6.4 (3.61-11.26)	8.01 (6.08-11.38)	0.012
Diabetes mellitus	53 (71.62%)	42 (66.67%)	0.580
Atrial fibrillation	8 (10.81%)	5 (7.94%)	0.771
NYHA class III-IV	51 (68.92%)	57 (90.48%)	0.003
CCS class			0.080
0	18 (24.32%)	23 (36.51%)	
II	22 (29.37%)	22 (34.92%)	
III	30 (40.54%)	13 (20.63%)	
IV	4 (5.41%)	5 (7.94%)	
Recent MI (≤ 90 days)	28 (37.84%)	24 (38.10%)	> 0.99
Old MI	61 (82.43%)	53 (84.13%)	0.823
Previous PCI	12 (16.22%)	14 (22.22%)	0.391
Troponin T (ng/ml)	0.038 (0.011-0.127)	0.024 (0.01-0.073)	0.367
Creatinine clearance (ml/min)	79 (55-95)	80 (64-106)	0.320
Bilirubin (µmol/L)	8 (6-13)	10 (7-14)	0.107

Continuous data were expressed as mean and standard deviation if normally distributed or median and (Q1- Q3) if non-normally distributed. Categorical data were expressed as numbers and percentages BMI=body mass index; CCS=Canadian Cardiovascular Society; EuroSCORE=European System for Cardiac Operative Risk Evaluation; MI=myocardial infarction; NYHA=New York Heart Association; PCI=percutaneous coronary intervention; TV=tricuspid valve

### Preoperative Echocardiographic Data

Patients in SVR and TV surgery group had significantly lower EF (*P*=0.010), higher LV diastolic dysfunction (*P*=0.005), higher PASP (*P*<0.001), higher end-systolic diameter (*P*=0.011), higher prevalence of preoperative MR grade 4 (*P*<0.001), and higher TR grade (*P*<0.001). RV basal dimension and RV dilatation were also higher in the TV surgery group (Table 2). RV dilatation was significantly associated with moderately severe TR (n=2; 100%) and severe TR (n=7; 70%) (*P*<0.001).

**Table 2:** Comparison of the preoperative echocardiographic data between surgical ventricular restoration patients with and without tricuspid valve surgery.

	No TV surgery (n=74)	TV surgery (n=63)	*P*-value
EF (%)	25 (20-30)	25 (20-25)	0.010
Aneurysmal apex	23 (31.08%)	15 (23.81%)	0.444
LV diastolic dysfunction	47 (64.38%)	50 (86.21%)	0.005
RWMA anterior wall			0.541
Normal	6 (8.11%)	2 (3.17%)	
Hypokinesia	6 (8.11%)	6 (9.52%)	
Akinesia	42 (56.76%)	33 (52.38%)	
Dyskinesia	20 (27.03%)	22 (34.92%)	
RWMA posterior wall			0.819
Normal	32 (43.24%)	26 (41.27%)	
Hypokinesia	27 (36.49%)	25 (39.68%)	
Akinesia	15 (20.27%)	11 (17.46%)	
Dyskinesia	0	1 (1.59%)	
RWMA inferior wall			0.904
Normal	33 (44.59%)	26 (41.27%)	
Hypokinesia	25 (33.78%)	23 (36.51%)	
Akinesia	16 (21.62%)	14 (22.22%)	
RWMA septal wall			0.501
Normal	36 (48.65%)	27 (42.86%)	
Hypokinesia	24 (32.43%)	20 (31.75%)	
Akinesia	14 (18.92%)	14 (22.22%)	
Dyskinesia	0	2 (3.17%)	
EDD (mm)	59.06±8.69 (n=71)	61.95±8.45 (n=63)	0.053
ESD (mm)	46 (40-53)	50 (45-55)	0.011
PASP (mmHg)	35 (30-45)	60 (45-70)	< 0.001
MR grade			< 0.001
No	12 (16.22%)	0	
Mild	19 (25.68%)	6 (9.52%)	
Moderate	24 (32.43%)	18 (28.57%)	
Moderate - severe	10 (13.51%)	8 (12.70%)	
Severe	9 (12.16%)	31 (49.21%)	
TR grade			< 0.001
No	44 (59.46%)	0	
Mild	25 (33.78%)	24 (38.10%)	
Moderate	5 (6.76%)	27 (42.86%)	
Moderate - severe	0	2 (3.17%)	
Severe	0	10 (15.87%)	
EDV (ml/m^2^)	164 (141.5-194)	170 (147-204)	0.411
ESV (ml/m^2^)	110 (93-141.15)	124 (101-155)	0.211
EDVi (ml/m^2^)	63.33 (50.16-82.83)	61.66 (49.66-72.4)	0.277
ESVi (ml/m^2^)	44.66 (34.16-59.16)	44.83 (34.66-52.66)	0.749
RV basal dimension	36.5 (33.5-40)	42.5 (37-48)	< 0.001
RV dilatation	6 (8.11%)	27 (42.86%)	< 0.001

Continuous data were expressed as mean and standard deviation if normally distributed or median and (Q1- Q3) if non-normally distributed. Categorical data were expressed as numbers and percentages EDD=end-diastolic diameter; EDV=end-diastolic volume; EDVi=end-diastolic volume index; EF=ejection fraction; ESD=end-systolic diameter; ESV=end-systolic volume; ESVi=end-systolic volume index; LV=left ventricular; MR=mitral regurgitation; PASP=pulmonary artery systolic pressure; RV=right ventricular; RWMA=regional wall motion abnormality; TR=tricuspid regurgitation; TV=tricuspid valve

### Operative Data

There were no differences in operative status, concomitant CABG, and the number of anastomoses between groups. Concomitant mitral valve replacement was more common in patients with TV surgery (*P*<0.001). Septal reshaping was the most common technique used in the TV surgery group (*P*=0.001), while septal reshaping and septoapical Dor procedure were performed more commonly in the no TV surgery group. Cardiopulmonary bypass and ischemic times were significantly longer in TV surgery patients (*P*=0.043 and *P*=0.026, respectively) (Table 3).

**Table 3:** Comparison of the operative data between surgical ventricular restoration patients with and without tricuspid valve surgery.

	No TV surgery (n=74)	TV surgery (n=63)	*P*-value
Emergency	13 (17.57%)	8 (12.70%)	0.483
CABG	71 (95.95%)	59 (93.65%)	0.703
Number of anastomoses	3.01±1.18	2.933±1.26	0.702
Total (n=130)	(n=71)	(n=59)	
Mitral valve surgery			< 0.001
Repair	41 (55.41%)	34 (53.97%)	
Replacement	9 (12.16%)	28 (44.44%)	
SVR type			0.001
Septal reshaping	24 (36.92%)	40 (63.49%)	
Septal exclusion (Guilmet)	10 (15.38%)	10 (15.87%)	
Septoapical (Dor)	24 (36.92%)	7 (11.11%)	
Inferior wall resection	1 (1.54%)	4 (6.35%)	
Lateral wall resection	6 (9.23%)	2 (3.17%)	
CPB (min)	136 (117-162.5)	154 (130-171)	0.043
Cross-clamping time (min)	109 (91-133.5)	126 (102-137)	0.026

Continuous data were expressed as mean and standard deviation if normally distributed or median and (Q1- Q3) if non-normally distributed. Categorical data were expressed as numbers and percentages CABG=coronary artery bypass grafting; CPB=cardiopulmonary bypass; SVR=surgical ventricular restoration; TV=tricuspid valve

### Postoperative Outcomes

Extracorporeal membrane oxygenation (ECMO) use was higher in SVR patients without concomitant TV surgery (*P*=0.015). TV surgery patients had significantly more re-exploration for tamponade and received more red blood cell (RBC) units (*P*=0.048 and *P*=0.037, respectively). There was no difference in other postoperative complications between groups. Hospital mortality occurred in eight (10.81%) patients who did not undergo TV surgery *vs.* five (7.94%) patients with TV surgery (*P*=0.771) (Table 4).

**Table 4:** Comparison of the postoperative data between surgical ventricular restoration patients with and without tricuspid valve surgery.

	No TV surgery (n=74)	TV surgery (n=63)	*P*-value
Open chest	9 (12.16%)	7 (11.11%)	> 0.99
IABP	12 (16.22%)	12 (19.05%)	0.822
CRRT/dialysis	10 (13.51%)	9 (14.29%)	> 0.99
ECMO	7 (9.46%)	0	0.015
Stroke	5 (6.76%)	0	0.062
Re-exploration for bleeding	7 (9.46%)	9 (14.29%)	0.431
Re-exploration for tamponade	1 (1.35%)	6 (9.52)	0.048
Re-exploration for another cause	8 (10.81%)	8 (12.7%)	0.793
Reintubation	24 (18.92%)	10 (15.87%)	0.660
Mechanical ventilation (hours)	11.55 (7.32-16.52)	13.75 (8.72-22)	0.157
Sepsis	9 (12.16%)	6 (9.52%)	0.785
Deep sternal wound infection	4 (5.41%)	2 (3.17%)	0.687
RBC transfusion	2 (1-4)	3 (2-5)	0.037
Fresh frozen plasma transfusion	4.5 (0-6)	4 (0-6)	0.750
Platelets transfusion	2 (0-6)	4 (0-6)	0.346
Atrial fibrillation	13 (17.57%)	13 (20.63%)	0.668
PPM	1 (1.35%)	1 (1.59%)	> 0.99
ICD	3 (4.05%)	1 (1.59%)	0.624
ICU stay (days)	3 (1-7)	4 (2-8)	0.686
Hospital stay (days)	13.5 (8-25)	15 (8-31)	0.574
Hospital mortality	8 (10.81%)	5 (7.94%)	0.771

Continuous data were expressed as mean and standard deviation if normally distributed or median and (Q1- Q3) if non-normally distributed. Categorical data were expressed as numbers and percentages CRRT=continuous renal replacement therapy; ECMO=extracorporeal membrane oxygenation; IABP=intra-aortic balloon pump; ICD=implantable cardioverter defibrillator; ICU=intensive care unit; PPM=permanent pacemaker; TV=tricuspid valve

### Long-Term Outcomes

Median follow-up time was 57 (21.57-99) months. Three patients required reintervention, one had TV repair (TV surgery group), and two had a mitral valve replacement (one from each group). Four patients had a stroke during follow-up, two from the SVR without TV surgery group and two from the TV surgery group.

**Fig. 1:**
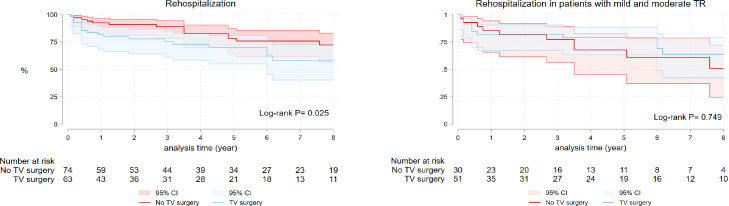
A) Kaplan-Meier curve for rehospitalization in surgical ventricular restoration patients with and without combined tricuspid valve (TV) surgery. B) Subgroup comparison of rehospitalization in patients with mild and moderate tricuspid regurgitation (TR). CI=confidence interval.

Cardiac rehospitalization occurred in 37 patients, 15 from the SVR without TV surgery group and 22 from the TV surgery group. Rehospitalization-free survival at one, three, five, and eight years was 92.45%, 88.92%, 78.21%, and 72.04%, respectively, for no TV surgery group and 81.56%, 72.76%, 69.90%, and 57.79%, respectively, for TV surgery group (log-rank *P*=0.025) (Figure 1A). There was no difference in rehospitalization in patients with mild and moderate TR from both groups (log-rank *P*=0.749) (Figure 1B). High PASP, low EF, and patients with no concomitant CABG were associated with increased rehospitalization (Table 5).

**Table 5:** Multivariable competing risk analysis for rehospitalization and recurrent tricuspid regurgitation and multivariable Cox regression for survival.

Rehospitalization	SHR (95% CI)	*P*-value
Tricuspid valve surgery	0.794 (0.315-1.998)	0.625
Coronary artery bypass grafting	0.342 (0.123-0.952)	0.040
Pulmonary artery systolic pressure	1.022 (1.000-1.044)	0.040
Ejection fraction	0.928 (0.875-0.985)	0.015
Recurrent tricuspid regurgitation	SHR (95% CI)	
TV surgery	0.30 (0.11-0.85)	0.023
Coronary artery bypass grafting	0.20 (0.07-0.58)	0.003
Ejection fraction	0.92 (0.85-0.98)	0.15
Pulmonary artery systolic pressure	1.03 (1.01-1.06)	0.013
Survival	HR (95% CI)	
Age, years	1.038 (1.003-1.074)	0. 030
Recent myocardial infarction	2.006 (1.006-4.000)	0.048
Bilirubin	1.034 (1.001-1.068)	0.038
Emergency	2.862 (1.349-6.073)	0.006
Tricuspid valve surgery	1.718 (0.844-3.495)	0.135
Cardiopulmonary bypass time (min)	1.008 (1.000-1.015)	0.035

CI=confidence interval; HR=hazard ratio; SHR=sub-distributional hazard ratio; TV=tricuspid valve

**Fig. 2:**
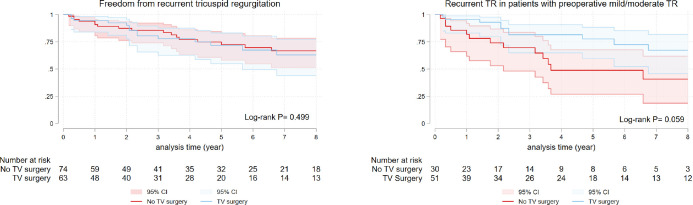
A) Kaplan-Meier curve for recurrent tricuspid regurgitation (TR) in surgical ventricular restoration patients with and without combined tricuspid valve (TV) surgery. B) Subgroup comparison of recurrent TR in patients with mild and moderate TR. CI=confidence interval.

Freedom from recurrent grade II or higher TR did not differ between groups (log-rank *P*=0.499) (Figure 2A). Freedom from recurrent TR in patients with preoperative mild or moderate TR was non-significantly higher in patients with no TV surgery (log-rank *P*=0.059) (Figure 2B). Factors associated with recurrent TR were low EF, high PASP, and TV surgery; CABG was protective (Table 5).

**Fig. 3:**
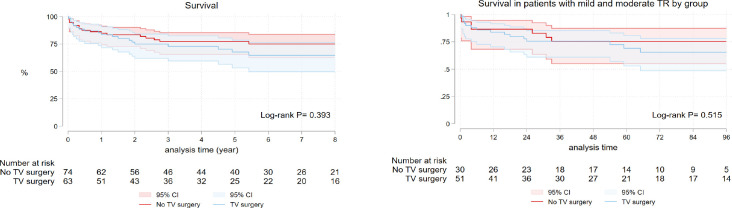
A) Kaplan-Meier survival curve in surgical ventricular restoration patients with and without combined tricuspid valve (TV) surgery. B) Subgroup comparison of survival in patients with mild and moderate tricuspid regurgitation (TR). CI=confidence interval.

Thirty-seven mortalities occurred during follow-up time - 18 in patients with no TV surgery and 19 in the TV surgery group. Survival at one, three, five, and eight years was 84.86%, 77.22%, 77.22%, and 74.95%, respectively, in no TV surgery group and 83.51%, 72.80%, 67.58%, and 64.64%, respectively, in the TV surgery group (log-rank *P*=0.394) (Figure 3A). There was no difference in mortality in patients with mild and moderate TR between groups (log-rank *P*=0.515). Factors affecting long-term survival were age, recent MI, high bilirubin level, emergency operation, and prolonged cardiopulmonary bypass time (Table 5).

### Echocardiographic Follow-Up

EF improved significantly after surgery in both groups (30.402±8.433% in the no TV surgery group and 28.507±6.951% in the TV surgery group) compared to the preoperative value (*P*<0.001 for both). EF in the TV surgery group was significantly lower compared to the no TV surgery group at any point during the follow-up (β: -2.64; 95% confidence interval [CI]: -5.044 - -0.249; *P*=0.030). However, change over time was not significant (β: 0.006; 95% CI: -0.007 - 0.019; *P*=0.383).

PASP was reduced significantly in both groups compared to the preoperative value. Postoperative PASP was 30.83± 9.21 in the no TV surgery group and 40.33±12.13 mmHg in the TV surgery group (*P*<0.001 for both). PASP in the TV surgery group was significantly higher compared to the no TV surgery group (β: 12.68; 95% CI: 8.45 - 16.91; *P*<0.001), while change over time was not significant (β: 0.039; 95% CI: -0.004 - 0.084; *P*=0.078).

RV dilatation in the TV surgery group was significantly higher compared to the no TV surgery group (β: 2.282; 95% CI: 1.427 - 3.136; *P*<0.001), and the change over time was not significant (β: 0.003; 95% CI: -0.0014 - 0.0141; *P*=0.113).

## DISCUSSION

This study explored the effects and long-term outcomes of combining TV surgery and SVR. In our study, patients in the TV surgery group were younger and had higher EuroSCORE, NYHA class III or IV, and lower EF. White et al. had conducted research on ventricular volume measurements to predict post-MI mortality rather than solely depending on the EF. The study showed five times higher post-MI mortality when the LV end-systolic volume index was > 60 ml/m^2^[^13]^. In SVR, reducing the LV size and maintaining a normal range of indexed LV end-systolic volume were targeted. It has shown better results when combined with coronary revascularization procedures^[13]^. Logically, increased volume will increase pressure, dilatation, and valvular regurgitation. The end-diastolic diameter was higher in our study in the TV surgery group, and we expect higher intraventricular pressure and significant ventricular dilatation. In our study, a significant dilatation was associated with higher grades of TR. The resultant dilatation can also be illustrated by the increase in annular diameter and MR, which explains our results of higher MR grades and concomitant mitral valve surgery that were noticed in SVR with TV intervention.

Several factors can indicate the need for TV surgical intervention, including the severity of the TR and PASP^[8]^. Those two factors were also higher in the TV surgery group. Different surgical interventions were attempted to improve heart function, including revascularization with CABG, correcting valvulopathies, partial LV resection, implanting assisting devices, or heart transplantation as an end-stage solution^[14]^. Prucz et al.^[6]^ conducted a study that showed combining revascularization and SVR had reduced long-term rehospitalization and improved long-term functional status. Although it was proved that surgical correction of mitral valve regurgitation would improve the abnormal ventricular geometry in ischemic cardiomyopathy patients with reduced EF, a study showed that SVR alone could restore normal ventricular architecture without mitral valve repair^[15]^. Partial LV ventriculectomy was associated with unsatisfactory results, including a high hospital mortality rate and unrecovered LV function[^16^].

Several techniques were identified for SVR procedures. Septal reshaping was proved to reduce LV volume and MR and to significantly improve functional NYHA class in about 72% of the studied patients^[17]^. Septal reshaping was the most commonly used technique in the TV surgery group. However, septoapical Dor technique was used at the same rate as septal reshaping in the group of patients without TV surgery. Repairing or replacing the TV would consume more operative time, which can explain the significantly longer ischemic and cardiopulmonary bypass times in the TV surgery group. We expect to achieve better hemodynamic status postoperatively after correcting concomitant valve lesions. Thus, SVR in the TV surgery group required a significantly lower ECMO use. Nevertheless, combining several surgical interventions may increase the operation complexity, complications, and the need for re-exploration. We have a significantly higher rate of postoperative re-exploration for tamponade and a higher number of transfused RBC units in SVR with TV surgery.

Concerning our long-term outcomes, the two groups were almost similar in terms of the need for further intervention and the incidence of stroke. Meanwhile, the rehospitalization rate was not significantly different between patients with mild or moderate TR in both groups. TR recurrence was insignificantly higher in patients with preoperative mild and moderate TR in the SVR without TV surgery group. A study that Lin et al. conducted found that recurrence of TR was associated with preoperative atrial fibrillation, severe TR, DeVega annuloplasty, postoperative permanent pacemaker insertion, and low preoperative EF, similarly to our findings^[18]^. Low EF and high PASP were risk factors for rehospitalization in our study. In addition, the lack of revascularization with CABG contributed to the overall incidence of rehospitalization. A research by Prucs et al.^[6]^ showed that combining CABG with SVR has a lower rehospitalization rate, estimated to be 24%, compared to the 55% rate in the group without CABG. Our study did not show any significant difference between the two groups in terms of mortality. This may indicate that combining TR surgery with SVR is a relatively safe practice.

In our research, both repair and replacement were considered in one group due to the limited number of cases. We suppose that having more cases and separating the two methods would give a better understanding of a better management strategy for concomitant TV disease during SVR.

### Limitations

The study is limited by the retrospective design with its inherent referral and selection biases. Moreover, this is a single-center study, and generalization of the results might be an issue. There are several risk factors that have affected the outcomes or patients’ selection and were not measured routinely.

## CONCLUSION

TV surgery concomitant with SVR is safe procedure, with similar operative mortality compared to the conservative approach. Concomitant TV surgery could reduce the recurrence of TR; however, its effect on the clinical outcomes of rehospitalization and survival was not evident. The same effect was observed in patients with mild and moderate TV regurgitation.
